# Origin of two-dimensional MXene/ferromagnetic interface evaluated by angle-dependent hard X-ray photoemission spectroscopy

**DOI:** 10.1080/14686996.2025.2551484

**Published:** 2025-08-22

**Authors:** Prabhat Kumar, Shunsuke Tsuda, Koichiro Yaji, Shinji Isogami

**Affiliations:** a Research Center for Magnetic and Spintronic Materials, National Institute for Materials Science (NIMS), Tsukuba, Japan; b Center for Basic Research on Materials, National Institute for Materials Science (NIMS), Tsukuba, Japan; c Unprecedented-scale Data Analytics Center, Tohoku University (UDAC), Tohoku University, Sendai, Japan

**Keywords:** Two-dimensional MXene, induced magnetic moment, HAXPES, XMCD

## Abstract

Emergent ferromagnetism on the surface of two-dimensional (2D) MXene is investigated by X-ray magnetic circular dichroism (XMCD) and angle-dependent hard X-ray photoemission spectroscopy (HAXPES). Focusing on Cr_2_N as one of the 2D-MXenes, high quality bilayers of Cr_2_N/Co and Cr_2_N/Pt are prepared by a magnetron sputtering technique. XMCD reveals the induced magnetic moment of Cr in the Cr_2_N/Co interface, while it is not observed in the Cr_2_N/Pt interface at room temperature. In order to distinguish the possible origins of either the interlayer magnetic exchange coupling or the charge transfer model as the source of ferromagnetism at the interface, the additional controlled Cr_2_N/Cu bilayer, whose work function of Cu is consistent with Co, is prepared. HAXPES spectra for the Cr 2*p* core level near the interface of Cr_2_N/Cu are consistent with that of Cr_2_N/Co, indicating that the induced magnetic moment of Cr observed by XMCD for Cr_2_N/Co can be attributed to the model of interlayer magnetic exchange coupling, rather than the charge transfer model, leading to emergent ferromagnetism at the interface with 2D-MXene.

## IMPACT STATEMENT

Induced magnetic moment is found on the surface of 2D-MXene by XMCD, and angle-dependent HAXPES analysis is used to show that it is not due to charge-transfer but can be attributed to the exchange magnetic coupling with the adjacent ferromagnetic layer.

## GRAPHICAL ABSTRACT

**Figure:**
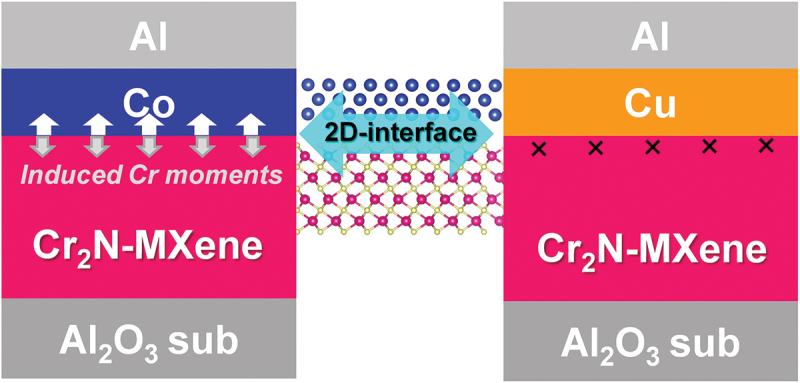

## 1. Introduction

Highly efficient semiconductor and/or magnetic devices are essential for the development of a modern society in which humans are connected to all kinds of applications via the Internet. In order to realize these devices, the reduction of the bit cell size towards the existing technology node and the reduction of the power consumption is the desired goal. Two-dimensional (2D) materials are considered as one of the candidates, not only because of their structural advantages but also because of various emergent phenomena at the interfaces of 2D heterojunctions, such as interlayer exchange coupling [1], magnetoelectric effect [2], and proximity effect [3,4].

One of the most attractive applications based on the emergent phenomena at interfaces is a spin-orbit torque (SOT) device, in which the spin current generated by an in-plane charge current flowing along a spin channel exerts torques on a ferromagnetic (FM) layer, resulting in SOT-driven magnetization switching [5]. While the conventional SOT device consists of the spin channel made of heavy metals such as W and FM layer [6], recent demonstrations have been conducted with the SOT devices consisting of 2D spin channels and 2D FMs which form a van der Waals type heterojunction, i.e. topological semimetallic WTe_2_/ferromagnetic Fe_3_GeTe_2_ [7,8]. There are two advantages of using the 2D spin channels, which are namely, the high spin injection efficiency due to the atomically flat interface, and the moderate change of electric potential at the interface due to the same Te termination, which causes the reduction of spin loss across the Fe_3_GeTe_2_ and WTe_2_ layers [9]. Furthermore, the 2D-Fe_3_GeTe_2_/Pt bilayer also exhibits the SOT-driven magnetization switching, although the interface does not form a van der Waals heterojunction [10]. This indicates that 2D materials have been promising for highly efficient SOT devices through interface engineering in the future.

MXene has recently become known as a new class of 2D materials [11]. The chemical formula is *M*_*n*+1_*X*_*n*_*T*_*x*_, where the sites *M*, *X*, and *T* represent transition metals such as Ti and Cr, 2*p* light elements such as C and N, and surface terminations such as O and Cl, respectively. Specifically, *n* corresponds to the number of *M-X-M* bonds, which varies from 1 to 4, and *x* is a variable. These parameters can effectively modulate the physical and chemical properties of MXene [12], because of the significant orbital hybridization between the elements *X* and *M*, which originates from the high electronegativity of the 2*p* light element *X* [13]. Thus, various applications have been demonstrated using MXenes in the fields of biomedicine [14], mechanical science [15], optoelectronics [16], and energy storage [17].

As mentioned above, emergent phenomena at interfaces are of increasing interest in 2D materials; however, it is not yet clear what phenomena occur at the 2D-MXene/FM interface. As an example, it is reported that the magnetic moment in nonmagnetic Cu and/or Pt can be induced by the adjacent FMs, and the origin is explained by both magnetic coupling and charge transfer [18,19]. If the magnetic moment of Cr in the MXene layer is induced by the adjacent FM layer, it would have a great impact on the spin injection efficiency for the spintronic devices with 2D systems [7,8], leading to a significantly important approach for efficient SOT-driven magnetization switching. The previous work had revealed that the induced magnetic moment appears at the interface between Cr_2_N and Co, but not at the interface between Cr_2_N and Pt [20]. At interfaces between materials with different work functions, charge transfer at the interface is inevitable, and this charge transfer has been proposed as a candidate origin of ferromagnetism. On the other hand, a mechanism involving exchange interactions has also been proposed, and no conclusive result had been reached, especially for the 2D materials, which has attracted considerable attention in both electronic engineering and physics.

In this study, we aim to analyze the induced magnetic moment and the electronic states of Cr in the Cr_2_N-MXene/Co bilayer, by means of both the X-ray magnetic circular dichroism (XMCD) and the angle-dependent hard X-ray photoemission spectroscopy (HAXPES). As a result, the magnetic moment of Cr was induced by Co, and the surface-sensitive HAXPES spectra of the Cr 2*p* core level were modulated by Co as well. The combined study with XMCD and HAXPES identified that the exchange magnetic coupling between Cr and Co is the main origin for the induced magnetic moment of Cr in the 2D MXene, rather than the charge transfer due to different work functions.

## 2. Experimental details

### 2.1. Film preparation and characterization

The Cr_2_N 2D-MXene was deposited on the *c*-plane oriented Al_2_O_3_ substrate using DC magnetron reactive nitridation sputtering with the Cr sputtering target. The substrate temperature (*T*_sub_) was varied from room temperature (RT) to 650°C, and the nitrogen flow ratio (*Q*) defined as *Q* = N_2_/(Ar + N_2_) was varied from 2% to 25% to form the stoichiometric Cr_2_N layer. The Co, Cu, and Al layers were deposited via DC magnetron sputtering at RT. The crystal structure was investigated via X-ray diffraction (XRD; SmartLab; Rigaku Corporation) with Cu-*K*_α_ radiation.

### 2.2. X-ray magnetic circular dichroism (XMCD) experiments

The XMCD measurements were performed at the BL14U Synchrotron Radiation Facility, NanoTerasu. Soft X-ray absorption spectra (XAS) were recorded at RT using the total electron yield (TEY) method with scanning photon energy. The XMCD signal was obtained as the difference of the XAS signal for circularly polarized light with positive and negative helicities. For the measurements of Cr and N, the XAS for each helicity was repeated five times and averaged to boost the signal-to-noise ratio. The magnetic field was applied perpendicularly to the surface of the sample.

### 2.3. X-ray photoemission spectroscopy (XPS) experiments

The conventional XPS of Al 2*s* core level was measured by the NIMS in-house system with the hemispherical analyzer (SES100, Scienta Omicron) and the excitation source of Al *K*_α_ without monochromator (DSX400, Scienta Omicron) at RT. The take-off angle (ToA) was varied at 90°, 60°, 30°, and 15° relative to the in-plane direction to adjust the surface sensitivity. The data acquired at ToA = 90° correspond to the normal emission configuration, which provides the highest bulk sensitivity configuration under the present experimental conditions.

### 2.4. Hard X-ray photoemission spectroscopy (HAXPES) experiments

The HAXPES experiment was performed at the BL09U Synchrotron Radiation Facility, NanoTerasu, using an incident synchrotron beam of photon energy ~6 keV. The photoemitted electrons were measured for two different ToAs of 10° and 88° with respect to the in-plane direction, that is, the photoemission from the surface is dominant for ToA = 10°, comparing to that for ToA = 88°. Using a monolithic Woltermirror, the incident X-ray beam was focused to 7 × 10 (vertical × horizontal) µm^2^. For the ToA = 88° condition, the footprint of the X-ray beam on the sample at grazing incidence was as small as 7 × 300 (vertical × horizontal) µm^2^. The photoelectrons were detected and analyzed using a high-resolution hemispherical electron analyzer (R4000, Scienta Omicron). All measurements were performed at RT. The high-brilliance synchrotron radiation allowed us to detect tiny differences in spectra, which are influenced by the adjacent layer. The energy axis of HAXPES spectra was calibrated based on the binding energy (*E*_b_) and the peak positions of Au 4*f*_7/2_ and Fermi level (*E*_F_) of Au.

## 3. Results and discussion

### 3.1. Crystal structure

**Figure 1.**
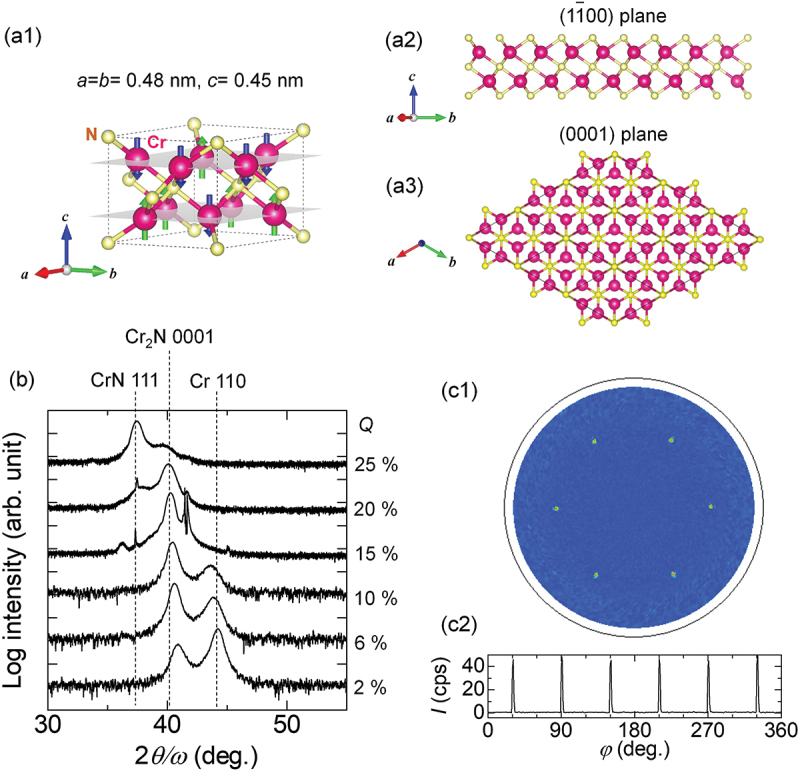
(a1–a3) Unit-cell model of Cr_2_N MXene together with possible magnetic structure (a1), cross-sectional plane view (a2) and top view (a3) of the Cr_2_N supercell. (b) Out-of-plane XRD profiles for 15-nm-thick Cr_2_N films with various nitrogen ratio relative to argon *Q*  = N_2_/(Ar+N_2_), while sputtering deposition on *c*-plane oriented Al_2_O_3_ substrates. (c1, c2) XRD Pole figure (c1) and φ-scan profile (c2) for the Cr_2_N ($\overset{ˉ}{1}\overset{ˉ}{1}21$).

Figure 1(a1) shows the unit cell of Cr_2_N MXene with a hexagonal structure, of which lattice constants are *a* = *b* = 0.48 nm and *c* = 0.45 nm. The collinear antiferromagnetic structure of Cr has been reported for a wide temperature range from 100 K to 500 K [21]. Figure 1(a2) and (a3) depict the supercell model of the Cr_2_N (1$\overset{ˉ}{1}$00) plane and (0001) plane, respectively. Figure 1(b) shows the out-of-plane XRD profiles for the 15-nm-thick Cr_2_N single layer depending on the N_2_ flow ratio, while reactive nitridation sputtering of Cr. Two XRD peaks appeared at 2θ/ω ≈ 42° and 44° for *Q* = 2%, suggesting coexisting of pure Cr and Cr_2_N phases. The XRD peak appearing at 2θ/ω ≈ 40° corresponds to the Cr_2_N (0001) plane, suggesting no coexisting phases for *Q* = 15%. Two XRD peaks appeared at 2θ/ω ≈ 37° and 40° for the *Q* higher than 20%, which correspond to the CrN (111) and Cr_2_N (0001) planes, respectively. Therefore, we determined the optimum *Q* for the single Cr_2_N MXene phase as *Q* = 15%. Figures 1(c1) and 1(c2) show the XRD pole figure and φ-scan profile for the Cr_2_N ($\overset{ˉ}{1}\overset{ˉ}{1}21$), suggesting the hexagonal structure with six-fold in-plane crystal symmetry in the Cr_2_N film.

### 3.2. XMCD to study induced magnetic moment of Cr

**Figure 2.**
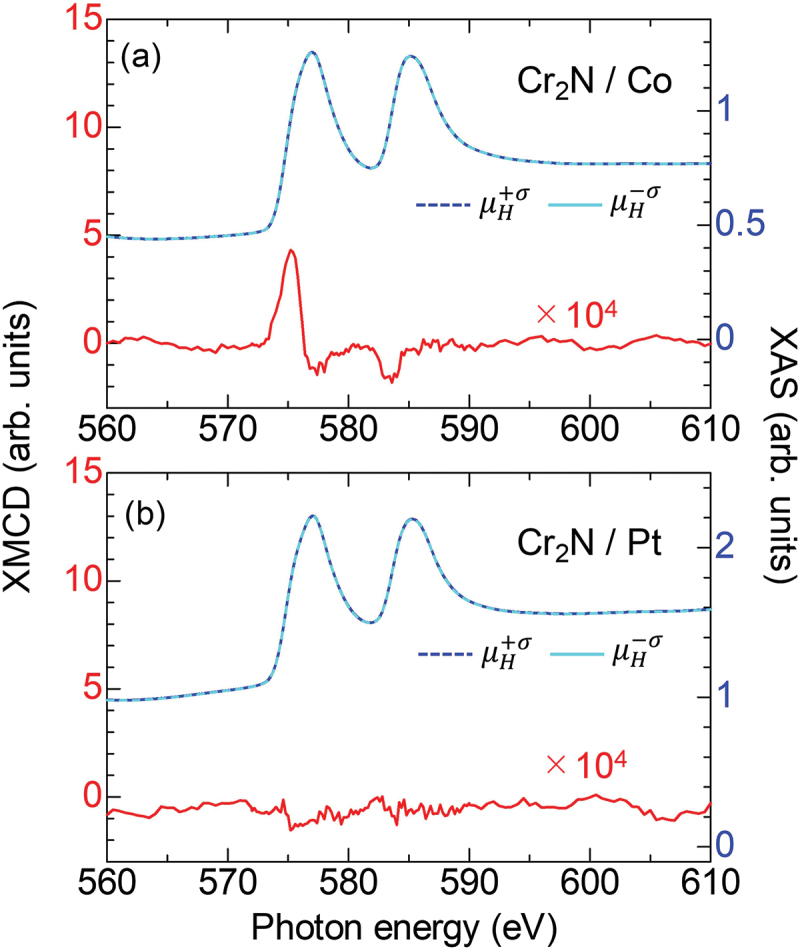
(a) XMCD (red) and XAS (blue) spectra for the Cr *L*_2,3_-edge of the sample, Al_2_O_3_ sub.//Cr_2_N(5 nm)/Co(1 nm)/Al(1 nm). (b) Same result as (a) but for the sample of Cr_2_N(5 nm)/Pt(1 nm). Modified from Kumar et al. (2025) ref [20], licensed under CC by 4.0.

We measured the element-selective magnetic properties at the Cr_2_N-MXene/FM interfaces by means of XMCD for two samples: (a) Cr_2_N(5 nm)/Co(1 nm), and (b) Cr_2_N(5 nm)/Pt(1 nm) [Figure 2(a, b)]. The X-ray absorption spectra (XAS) near the *L*_2,3_-edge of Cr exhibited two peaks. The XMCD signal was evident near the Cr *L*_2_-edge for Cr_2_N/Co, while it was not observed for Cr_2_N/Pt. Using the sum rule (see Figure S1 in the Supporting Information) for the results of the Cr_2_N/Co bilayer, spin (*m*_spin_) and orbital (*m*_orb_) magnetic moments of Cr were estimated to be −0.063 *µ*_B_ and ~0 *µ*_B_, respectively. The magnitude of induced moment of Cr is 25 times smaller than that of the calculated value of Co (*m*_spin_ ≈ 1.63 *µ*_B_ and *m*_orb_ ≈ 0.1 *µ*_B_) [22]. The *m*_spin_ of Cr corresponds to the uncompensated moment ($m_{Cr}^{UC.}$) originating from the imbalance in the antiferromagnetic structure of Cr_2_N due to the adjacent Co layer.

### 3.3. XPS to study the surface oxidation state

**Figure 3.**
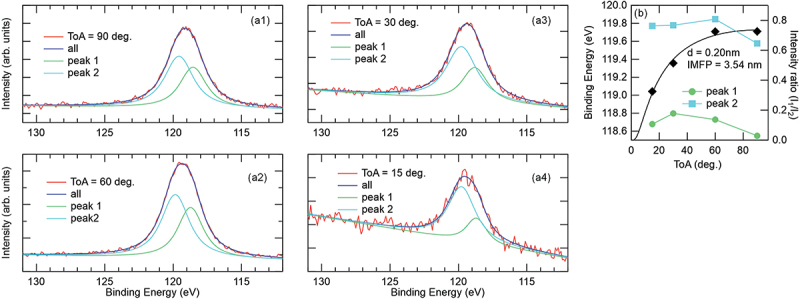
(a1–a4) XPS spectra of Al 2*s* core level for the sample, Al_2_O_3_ sub.//Cr_2_N(5 nm)/Al(1 nm), with ToA of 90°, 60°, 30°, and 15°, where the spectra were fitted by two peaks, which are the peak 1 and peak 2 originating from the metallic Al and Al-O, respectively. Note that the spectra with lower ToA detects more photoelectrons from top surface. (b) ToA dependences of binding energy and intensity ratio (*I*_1_/*I*_2_), where the *I*_1(2)_ denotes integral intensity of the peak 1(2).

To investigate the surface oxidation state of the 1-nm-thick Al film used as a capping layer, angle-dependent XPS was employed to analyze the Al 2*s* core level. Figures 3(a1-a4) show the XPS spectra for the Cr_2_N(5 nm)/Al(1 nm) with various ToA. Notably, spectra acquired lower ToA detect more photoelectrons from the top surface. The spectra were reproduced with two peaks, namely, peak 1 and peak 2 that are originating from metallic Al and Al-O, respectively. Figure 3(b) shows the ToA dependences of *E*_b_ and intensity ratio (*I*_1_/*I*_2_), where the *I*_1(2)_ denotes the integral intensity of the peak 1(2). *E*_b_ for peak 1 slightly decreased with increasing ToA, which was consistent with that for peak 2. The reason for this shift is unknown, but the shift amount is ~0.2 eV, which is sufficiently smaller than the energy resolution (~1 eV). In addition, the *I*_1_/*I*_2_ increased monotonically from ~0.32 to ~0.73 with increasing ToA, indicating that the metallic Al component is more dominant at higher ToAs compared to Al-O, which is more pronounced at lower ToA. The superimposed curve was obtained from calculation based on Ref [23]. The inelastic mean free path was derived from Ref [24], assuming inorganic materials. The calculated result closely reproduced the experimental result, yielding a surface oxide thickness of ~0.2 nm. We thus conclude that the Al-O was present only near the surface of the Al capping layer, which was estimated to be ~0.2 nm. It is inferred that the natural oxidation of the Cr_2_N surface can be ruled out from the consideration of HAXPES spectra shown below.

### 3.4. HAXPES to study the electronic state of Cr

**Figure 4.**
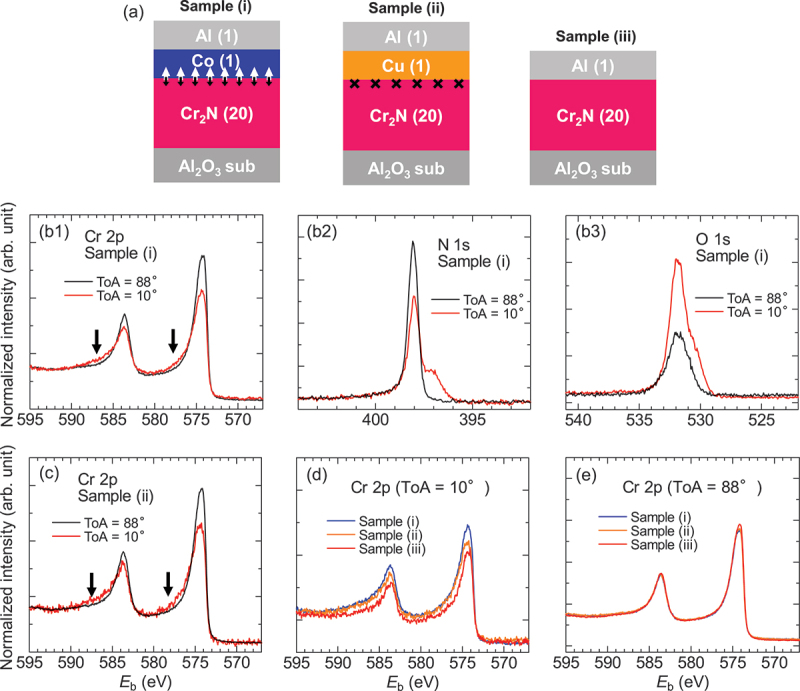
(a) Sample variations to investigate the electronic state of Cr affected by the adjacent Co [sample (i)], Cu [sample (ii)], and Al layers [sample (iii)]. The number in parentheses represents the layer thickness. (b1–b3) HAXPES spectra for the Cr 2*p* core level (b1), N 1*s* core level (b2), and O 1*s* core level (b3) of the sample (i). Black and red lines correspond to the spectra with ToA = 88° and 10°, respectively. (c) Same measurements as (b1) but for the sample (ii). (d,e) comparison among the spectra for ToA = 10° (d) and that for ToA = 88° (e).

Figure 4(a) shows the film stacking structure to evaluate the electronic states of Cr influenced by the adjacent Co [sample (i)], Cu [sample (ii)], and Al [sample (iii)]. Note that the Cr_2_N/Co and Cr_2_N/Cu interfaces do not have intermixing and/or alloying, judging from the X-ray reflection analysis (see Figure S2 in the Supporting Information). The crystal orientation of the fcc-Cu at the interface is (111), because the interface of the Cr_2_N layer is terminated by the (0001) plane, which is the equivalent crystal plane to the fcc-(111) plane. In-plane lattice constant of Cu and Cr_2_N are 0.51 nm and 0.48 nm, respectively, which corresponds to a mismatch of 6.3%. Therefore, the fcc-Cu layer can epitaxially grow with (111) orientation on the Cr_2_N layer in the sample (ii). These samples were prepared for the following purposes. First, comparison between samples (i) and (ii) allows for separating the possible origins of $m_{Cr}^{UC.}$, such as the magnetic coupling effect by the adjacent Co layer or the charge transfer by different work functions (WF), because the WF of Co is ~5.0, which is consistent with that of (111) plane-oriented Cu [25]. Namely, the origin of $m_{Cr}^{UC.}$ can be determined as the magnetic coupling effect, when no change in HAXPES spectra was observed between samples (i) and (ii). Second, sample (iii) provides the electronic states of Cr in the pristine Cr_2_N MXene.

Figure 4(b1) shows the HAXPES spectra for the sample (i) with the ToA of 10° and 88°. Spectral normalization was performed outside the effective energy range, on both the high-binding energy and low-binding energy sides. The subsequent spectra in Figure 4(b2, e) were normalized using the same procedure. The peaks appeared at the *E*_b_ of 584 eV and 574 eV, which correspond to the Cr 2*p*_1/2_ and 2*p*_3/2_ core levels, respectively. Each peak exhibited two components: a main peak and a satellite at a higher binding energy, as indicated by the arrows. With the present normalization, the intensity of the main peak at ToA = 88° was larger than that at ToA = 10°. In contrast, the satellite was more prominent at ToA = 10°. Cr_2_N layer is located away from the sample surface. Therefore, photoelectrons must travel a certain distance within the sample before being emitted into the vacuum. At ToA = 10°, the flight distance is longer, resulting in more photoelectrons being scattered. As a result, the information from regions closer to the interface is more dominantly represented at ToA = 10°. On the other hand, at ToA = 88°, photoelectrons are emitted at nearly the shortest distance, so more information from the interior of the film is preserved compared to ToA = 10°. In Figure 4(b1), the main peak is strong at ToA = 88°, and the satellite peak is strong at ToA = 10°, suggesting That the main peak represents the bulk film dominantly at ToA = 88°, whereas the satellite is associated with the interface of sample (i).

Note that the intensity variation of the satellite, as indicated by arrows, suggests that the electronic state of Cr_2_N was modulated by the adjacent Co layer. Figures 4(b2, b3) show corresponding results for sample (i) for the N 1*s* and O 1*s* core levels, respectively. The hump-like feature appeared at lower *E*_b_ than 1*s* main peak at ToA = 10°, whereas no such feature was observed at ToA = 88°. This can be attributed to the bonding state of N with Co at the Cr_2_N/Co interface. The intensity of the O 1*s* core level was much higher at ToA = 10°, comparing to the case of ToA = 88°. This result is consistent with the surface oxidation of the Al capping layer, as evaluated by conventional XPS in Section 3.3

To investigate the effect of the magnetic moment in the Co layer to the Cr_2_N layer, the same measurements were conducted on the sample (ii), in which the Cr_2_N layer has an interface with a Cu layer instead of a Co layer. Figure 4(c) presents the Cr 2*p* core level spectra of sample (ii). We can see the same results, that is, a prominent main peak appeared at ToA = 88°, while the satellite is more prominent at ToA = 10°. Thus, the electronic state of Cr_2_N was modulated by the Cu layer, resulting from the bonding state between N and Cu. By comparing the spectra of the samples (i), (ii), and (iii) for ToA = 10°, it was revealed that the electronic state of Cr near the interface remained consistent regardless of whether the adjacent layer was Co or Cu, as shown in Figure 4(d). The spectra for sample (iii) show smaller intensity at the *E*_b_ from 573 eV to 590 eV. On the other hand, the discrepancy becomes negligible for samples (i), (ii), and (iii) with ToA = 88°, as shown in Figure 4(e), indicating that the discrepancy is confined to the interface. Note that these results are also confirmed in the N 1*s* core levels of samples (i), (ii), and (iii) (see Figure S3 in the Supporting Information).

As a discussion, we consider the observed XMCD signal as shown in Figure 2. The induced magnetic moment of Cr, $m_{Cr}^{UC.}$, was evident by the adjacent Co layer at the Cr_2_N/Co interface, while it was not observed at the Cr_2_N/Pt interface, in which the WF of Co (~5.0) is smaller than that of Pt (~5.7). To exclude the influence of the different WF, we replaced the Pt with Cu, of which WF is similar to that of Co. As shown in Figure 4(d), the interfacial electronic state of Cr_2_N/Co is consistent with that of Cr_2_N/Cu, which confirms that the interlayer magnetic coupling is one of the major origins for the $m_{Cr}^{UC.}$ in the Cr_2_N 2D-MXene, rather than the charge redistribution. Based on these results, we thus infer that the $m_{Cr}^{UC.}$ is not expected for the Cr_2_N/Cu interface by the XMCD measurements. Within the framework of 2D ferromagnetism, van der Waals ferromagnetic 2D materials have recently attracted considerable attention due to their potential for practical applications. For example, Fe_3_GaTe_2_ exhibits intrinsic ferromagnetism with Curie temperature above RT and sizable perpendicular magnetic anisotropy [26]. This is mostly originating from the electronic state of 3*d* orbitals of transition metals that are modulated by the various lattice symmetry such as honeycomb and triangle structures, which is classified as the intrinsic ferromagnetism of pristine 2D materials. On the other hand, some reports show emergent and/or tailored 2D magnetism with pressure and elemental doping [27,28], which is classified as the extrinsic one. In addition, magnetic proximity effect has been examined in 2D van der Waals heterojunction systems [29,30]. Although various mechanisms are reported mentioned above, emergent ferromagnetism due to surface termination is unique characteristics for MXene, e.g. F- and OH-terminated Cr_2_C and/or Cr_2_N are predicted to be ferromagnetic [31]. Therefore, the findings in this study show that the magnetic exchange coupling in the MXene/FM system could open another pathway to induce the ferromagnetism in the MXene, leading to a phenomenon of spin-filtering effect in the 2D devices [32].

## 4. Conclusion

To examine the origin of the induced magnetic moment of Cr, $m_{Cr}^{UC.}$, in the Cr_2_N MXene/Co bilayer, we evaluated interfacial electronic states via angle-resolved HAXPES at RT with sufficient sensitivity at the NanoTerasu synchrotron radiation facility. The controlled Cr_2_N-MXene/Cu was prepared for comparison, which of these samples allow us to distinguish the possible major origins, that is, the interfacial magnetic coupling and the charge transfer. The interface sensitive HAXPES spectra for Cr_2_N/Co were consistent with that for Cr_2_N/Cu. Furthermore, bulk sensitive ones for both samples were consistent. These results led us to conclude that the inter-layer magnetic coupling can be a major origin for the induced magnetic moment in the 2D MXene adjacent to FM, rather than charge transfer due to the different WFs.
